# Influence of Ageing on Optical, Mechanical, and Thermal Properties of Agricultural Films

**DOI:** 10.3390/polym15173638

**Published:** 2023-09-04

**Authors:** Maja Rujnić Havstad, Ines Tucman, Zvonimir Katančić, Ana Pilipović

**Affiliations:** 1 University of Zagreb Faculty of Mechanical Engineering and Naval Architecture, Ivana Lučića 5, 10000 Zagreb, Croatia; ines.tucman@fsb.hr (I.T.); ana.pilipovic@fsb.hr (A.P.); 2 University of Zagreb Faculty of Chemical Engineering and Technology, Trg Marka Marulića 19, 10000 Zagreb, Croatia; katancic@fkit.unizg.hr

**Keywords:** ageing, polyethylene, FT-IR, DMA, DSC, ethylene tetrafluorethylene, tensile properties, TGA, transmittance

## Abstract

Plastic films utilized as greenhouse coverings play a vital role in safeguarding plantations from diverse weather conditions like sunlight, rain, hail, and wind. It is essential for these films to preserve their properties even after extended exposure to sunlight and water, while also maintaining transparency to support the unhindered growth of plants. The purpose of the study was to compare the properties of three types of plastic films: low density polyethylene diffuse film, low density polyethylene clear film, and ethylene tetrafluoroethylene film, before and after their ageing in weather test chamber with xenon-arc light in the presence of moisture. Two distinct types of PE films were chosen based on their suitability for specific regions in Croatia, whereas ETFE film was chosen as a potential new material that is gaining popularity across various industries, including agriculture. The properties investigated were tensile properties, transmittance by spectral analysis, and viscoelastic properties by dynamic mechanical analysis. Also, untreated films and the ones exposed to artificial ageing were compared by means of Fourier-transform infrared spectroscopy, differential scanning calorimetry and thermogravimetric analysis. The administered tests revealed a certain level of property degradation due to ageing in all three films. However, none of the films showed a substantial level of deterioration, indicating their suitability as greenhouse coverings.

## 1. Introduction

Greenhouse covers have a profound impact on the environmental conditions experienced by crop plants cultivated within these enclosures. The primary effect is their ability to partially isolate the interior from the external atmosphere, resulting in significant changes to various factors crucial for plant growth [1].

During usage, plastic materials undergo ageing, leading to a decline in their performance and eventual loss of utility. When exposed to natural elements such as light, heat, oxygen, and water, plastics can be significantly affected. This exposure can cause the surface of plastics to yellow or crack, and their overall properties degrade over time. Consequently, the useful life of the plastic is shortened because of these deteriorative effects [2].

Exposure to solar radiation and chemical products during cultivation can cause degradation of greenhouse plastic films. The service life of these films depends on their composition, with polyolefin materials, such as low-density polyethylene (LDPE), and ethylene-vinyl acetate (EVA) copolymers, lasting anywhere from a few months up to 3 to 4 years, depending on the film’s thickness and degree of stabilization. In contrast, ethylene tetrafluoroethylene (ETFE) copolymer films have a significantly longer service life, due to their inherent UV stability, which eliminates the need for any additional UV stabilizers. Additionally, these copolymer films exhibit low chemical reactivity towards commonly used agrochemicals, contributing to their extended lifespan compared to other materials [3].

The most exploited materials belong to the LDPE group, known for their versatility in accommodating additives that enhance optical clarity, thermal performance, and overall durability. Long-life PE, derived from resins with low-fluidity indices and high degrees of polymerization, incorporates stabilizers as additives to protect against the damaging effects of ultraviolet (UV) rays. To further enhance the thermal properties of these plastics, a typical approach involves copolymerizing ethylene with ethylene vinyl acetate (EVA) [4]. Low-density polyethylene (LDPE) film is widely used as a greenhouse covering material in the Mediterranean region, primarily due to its affordability, remarkable mechanical attributes, and exceptional resistance to chemicals. However, exposure to weathering conditions, particularly solar irradiation within the 290–400 nm range, impacts the chemical structure of LDPE film, leading to changes in its mechanical and physical properties [5].

The research of Dilara and Brassoulis [6] demonstrated that the degradation of LDPE films employed as greenhouse coverings is a complex process involving various interconnected mechanisms. These include photo-degradation triggered by reactions facilitated by UV-irradiation, chemical degradation resulting from interactions with air pollutants and agrochemicals, and eventually, mechanical degradation due to bond ruptures induced by mechanical stress. 

As reported by Briassoulis in [5], the most harmful form of degradation affecting LDPE films is caused by UV radiation, commonly known as photo-degradation. This process involves reactions that alter the polymer’s primary structure through chain scission, crosslinking, and oxidative processes. Additionally, the degradation rate of LDPE greenhouse films can be further accelerated by a combination of various factors, including temperature, agrochemicals, mechanical stress, and other relevant parameters, in conjunction with UV radiation. These findings were supported by Han et al. [7], who concluded that both photo- and thermo-oxidation processes lead to concurrent occurrences of chain scission, cross-linking, and branching, giving rise to several degradation products, such as carbonyl groups.

Polyethylene is a polymer with both crystalline and amorphous regions. Chen et al. discovered that the degradation of plastic films starts in amorphous regions before the crystalline regions [8]. The UV radiation generates free radicals, which react either with oxygen to cause chain scission or with each other, resulting in crosslinking [9].

Antioxidants play a crucial role in protecting the PE film from the detrimental effects of UV radiation and heat over extended periods. A wide array of additives are presently accessible, which, when incorporated in small quantities into polymers, enhance their stability when exposed to heat and UV radiation [10].

Babaghayou et al. studied chemical and mechanical stability, as well as the anisotropy of properties for LDPE greenhouse covering films, as a function of weathering time. Natural weathering also induced structural changes, with higher exposure times leading to increased crystallinity degree, crystal thickness, and birefringence [11]. 

The physical and mechanical properties of polyethylene films utilized as greenhouse covers can be influenced by three primary categories of conditions or factors. According to Dehbi et al. [12], these categories include: (a) product manufacturing and process specifications, (b) greenhouse external climate conditions, and (c) greenhouse microclimate (internal) conditions. 

Abdelhafidi et al. in [13] uncovered that polyethylene undergoes a photooxidation process, reacting with the surrounding oxygen. The study highlights that crosslinking and chain scissions are the predominant events occurring during ageing. These two essential reactions irreversibly alter the average molecular weight, significantly impacting the mechanical properties of the material and consequently reducing the films’ service life.

Luyt et al. observed changes in the tensile properties (such as increased Young’s modulus and decreased elongation at break), which suggested a chain scission/branching mechanism that led to crosslinking for LDPE [14].

ETFE, a less commonly used material for greenhouse covering compared to LDPE, is a copolymer of ethylene and fluoroethylene. This versatile material finds application in various fields, due to its lightweight nature, high durability, and self-cleaning capability. ETFE can be extruded into films, which are adaptable for both single and multi-layer cladding purposes. The films usually come in thicknesses ranging from 50 μm to 300 μm. One of its notable features is its impressive light transmission rate, with a high 90% transmission rate for visible light and 83% for infrared light. These attributes make ETFE film membrane a sought-after choice for large membrane structures as it can provide a safe, long-lasting, and visually appealing solution for construction projects [15,16]. 

Stefani et al. in [3] detected that even after an artificial ageing of 9800 h, the ETFE film sample exhibited minimal changes, indicating its remarkable stability and resistance to degradation. This confirmed Bracciale et al. in [17], where the results showcased the exceptional properties of ETFE after artificial ageing. In the review conducted by Lamnatou et al., it was concluded that ETFE material exhibits resistance to temperature/ageing, possesses high mechanical strength, and demonstrates excellent chemical resistance [18]. Because of their durability, ETFE films also contribute to generating less waste. By analysing the waste produced during the lifespan of greenhouse coverings, Maraveas in [19] estimated their environmental impact. A comparison of the waste generated by LDPE, EVA, and ETFE revealed that LDPE and EVA result in the highest accumulated waste quantity when considering a 15-year greenhouse lifespan. Consequently, it can be concluded that ETFE plastic films have the least environmental impact compared to both EVA and LDPE options.

However, the absence of comprehensive understanding about the effect of ageing on the films used as greenhouse coverings has been noted, and the objective of this study is to address this gap. Two types of PE films were chosen for their predominant use as greenhouse coverings in Croatia: diffuse films and clear films. Additionally, ETFE was chosen as a promising new material that could potentially replace clear polyethylene film, offering a range of advantageous properties. PE films have been extensively utilized as greenhouse coverings for a considerable period, leading to comprehensive studies of their properties. However, the same cannot be said for ETFE, particularly in the context of its potential use in agriculture. Therefore, we decided to carry out the comprehensive comparison of the properties of all three types of films using several investigative methods, which include testing of tensile properties, spectral analysis, dynamic mechanical analysis, Fourier-transform infrared spectroscopy, differential scanning calorimetry, and thermogravimetric analysis.

## 2. Materials and Methods

### 2.1. Materials

In the coastal part of Croatia, diffuse PE films are predominantly used as greenhouse coverings. These films are designed to spread light more evenly and reduce direct sunlight exposure, making them well-suited for the coastal climate. In the continental part of Croatia, clear PE films are more commonly employed as greenhouse coverings. Clear films allow more direct sunlight to penetrate, which can be beneficial in regions with cooler temperatures and less natural sunlight [20]. The choice of PE films in each region is based on their specific climate conditions and the desired light diffusion requirements for optimal plant growth in the respective areas. Three different types of films were selected for the experiments, and they were tested before and after they were exposed to ageing: ETFE, transparent/clear PE (PE-C), and diffuse PE (PE-D). The purchased polyethylene films were intended to be used as greenhouse coverings. The thickness of the ETFE film was 0.1 mm, while both PE films had a thickness of 0.2 mm.

### 2.2. Artificial Ageing

Ageing was carried out in a *Cofomegra Solarbox RH* (Milan, Italy) xenon test chamber (that has xenon-arc light in the presence of moisture, so there is a possibility of regulating temperature, humidity, and/or wetting.

Weather ageing chamber conditions according to ISO-4892-2:2013: Plastics—Methods of exposure to laboratory light sources—Part 2: Xenon-arc lamps [21] are:

- Irradiation: 550 W/m^2^,
- Temperature: 65 °C and humidity 65% for 1000 h, which roughly corresponds to 3 years of actual ageing exposure to atmospheric conditions,
- Dry time/wet time exposure alternated in duration of 102 and 18 min, respectively.

### 2.3. Differential Scanning Calorimetry (DSC)

The thermal properties of the polymer films studied were determined using a *Mettler Toledo DSC 3* (Columbus, OH, USA) differential scanning calorimeter (DSC). Samples weighing about 5 mg were heated from −80 °C to 300 °C to delete thermal history, followed by a cooling cycle to −80 °C and reheating. To obtain 5 mg of the sample, several pieces of film were cut to fit the crucible. The measurement was performed in a nitrogen atmosphere (N_2_) at a flow rate of 50 mL/min and a heating rate of 10 °C/min. The enthalpy of melting (Δ*H*_m_) and crystallization (Δ*H*_c_), crystallization temperature (*T*_c_), and melting temperature (*T*_m_) were determined.

### 2.4. Testing of Tensile Properties

Testing of tensile properties was performed on a *Shimadzu AGS-X* (Tokyo, Japan) universal testing machine, according to standard ISO 527-3:2018: Plastics—Determination of tensile properties—Part 3: Test conditions for films and sheets [22]. The speed of testing was 100 mm/min. The examination was conducted on three test specimens, followed by the computation of the mean value and standard deviation.

### 2.5. Spectral Analysis

The transmittance measurement procedure at different wavelengths was performed on a *Thermo Scientific Evolution 350* (Waltham, MA, USA) UV–VIS spectrometer with a source of electromagnetic radiation in the range 190 nm to 1100 nm in the following way:

- The plastic films were cut into small pieces using a die-cutter.
- The pieces of films were placed on a glass plate and covered with another piece of glass.
- The UV–VIS spectrometer was turned on, and the transmittance mode was selected.
- A blank was created by placing a piece of blanket on the machine.
- The blank was subtracted from the readings of the samples.
- The data was collected for both groups of samples, with and without weather chamber ageing.

The test was carried out on 6 test specimens.

### 2.6. Fourier-Transform Infrared Spectroscopy (FT-IR)

The polymer films studied were characterized with attenuated total reflectance Fourier transform infrared spectroscopy (ATR FTIR) using a *Perkin Elmer Spectrum One* (Waltham, MA, USA) FTIR spectrometer equipped with ZnSe crystal in the range 4000 to 650 cm^−1^ with a resolution of 4 cm^−1^. Each spectrum was an average of three spectra recorded at different positions on the film. Films were previously wiped to remove any surface contaminants.

### 2.7. Dynamic Mechanical Analysis (DMA)

Dynamic mechanical analysis (DMA) is a technique that is used to characterize polymers’ properties as a function of temperature, time, frequency, stress, or a combination of all these parameters. Dynamic mechanical analyser *DMA 983*, manufactured by *TA Instruments* (New Castle, DE, USA), was used to measure the primary viscoelastic functions, storage modulus (*E*′), and loss modulus (*E*″). The measurements were performed at a constant frequency of 1 Hz with amplitude of 0.1 mm in the stretching mode. The heating rate was 3 °C/min, and the temperature range was from −100 °C to 120 °C. Liquid nitrogen was used for cooling at low temperatures.

### 2.8. Thermogravimetric Analysis (TGA)

Thermogravimetric analysis (TGA) of the studied samples was performed using a *Mettler Toledo TGA/DSC 3+* (Columbus, OH, USA). Results were obtained for samples weighing about 5 mg in the temperature range of 35 °C to 600 °C, at heating rates of 10 °C/min under N_2_ atmosphere with a constant flow rate of 50 mL/min during the analysis. Sample preparation was identical to that described for DSC. The residual yield was determined (*m*_600_), as well as the temperature at which 5 mass percent of the sample was decomposed (*T*_95_) and the temperature at the maximum decomposition rate (*T*_max_).

## 3. Results and Discussion

### 3.1. Melting Points and Crystallinity (DSC)

**Figure 1:**
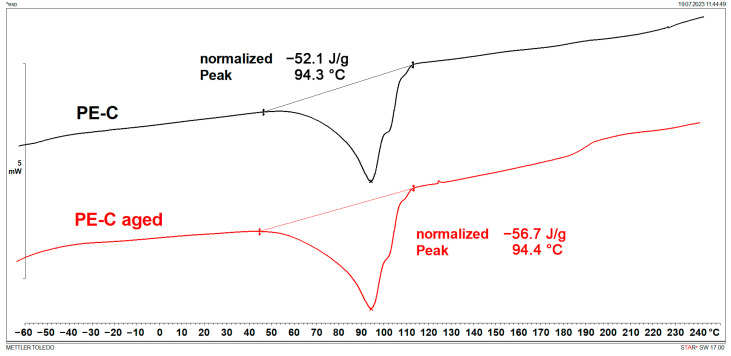
DSC heating curves of PE-C.

**Figure 2:**
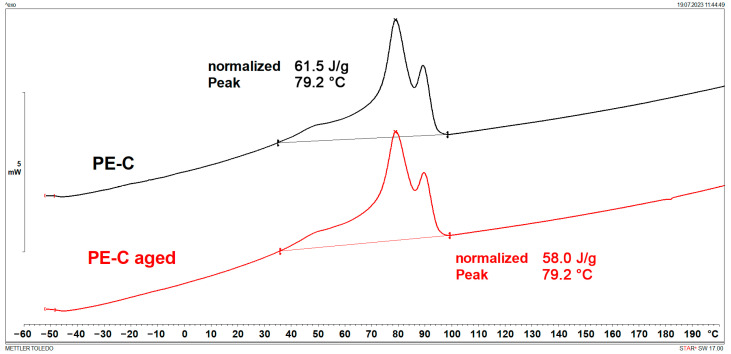
DSC cooling curves of PE-C.

**Figure 3:**
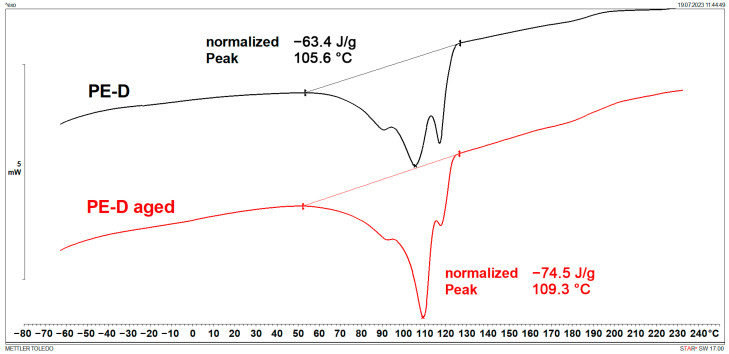
DSC heating curves of PE-D.

**Figure 4:**
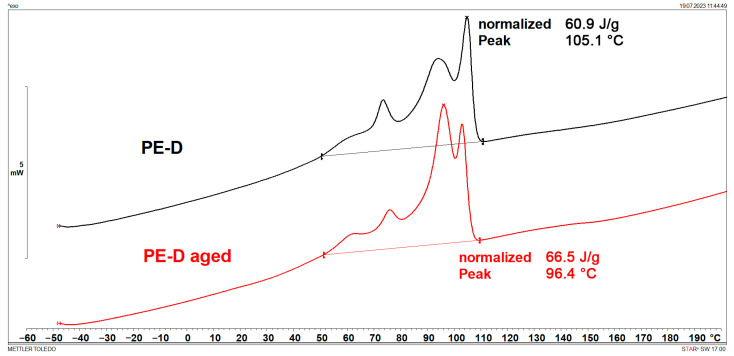
DSC cooling curves of PE-D.

**Figure 5:**
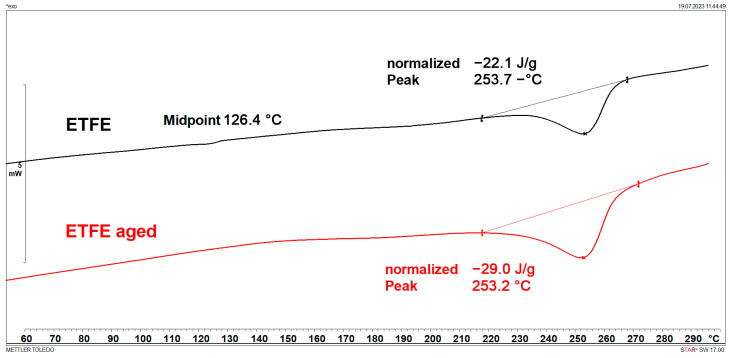
DSC heating curves of ETFE.

**Figure 6:**
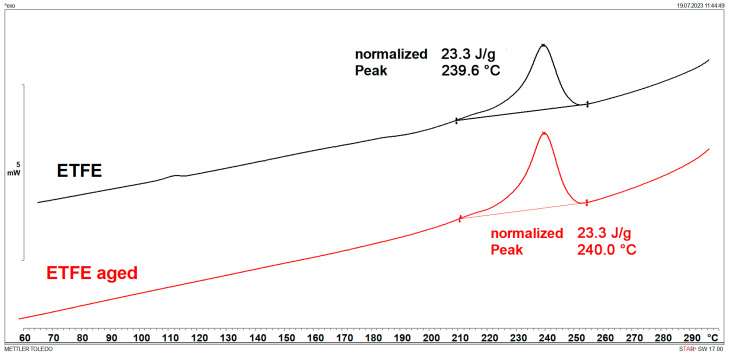
DSC cooling curves of ETFE.

DSC results are shown in Figure 1, Figure 2, Figure 3, Figure 4, Figure 5 and Figure 6 and Table 1.

Figure 1, Figure 2, Figure 3 and Figure 4 display multiple peaks observed during the melting and crystallization processes of the PE-C and PE-D samples, indicating the presence of crystallites of different sizes in the polymer. Ageing does not affect the melting and crystallization temperatures of PE-C, but it affects melting enthalpy, which increased by about 4.5 J/g. By dividing the enthalpy of investigated samples studied with the melting enthalpy of 100% crystalline polyethylene of 293 J/g as it was explained by Wunderlich in [23], the degree of crystallinity can be calculated, and this increase in enthalpy corresponds to an increased crystallinity of about 1.5%. According to Chen et al. [24], this indicates a decrease in the molecular weight of PE after ageing, which allows for easier stacking of shorter polymer chains into crystallites. In general, both the overall growth rate of crystallization and the resulting crystallinity increase with decreasing molecular weight. Such behaviour is consistent with some other papers dealing with ageing of polymer materials, where an increase in crystallinity was regularly observed as a consequence of decrease in molecular weight [25,26]. In the case of PE-D, ageing affects both the melting temperature, which increases by about 4 °C, and the crystallization temperature, which decreases by about 9 °C. The increase in crystallinity is even more pronounced than for the PE-C sample, as it increased by 4%. Finally, for ETFE (Figure 5 and Figure 6), there is no change in *T*_m_ and *T*_c_, but in this case the increase in crystallinity is about 6%, indicating the strongest chain shortening. The crystallinity was calculated from the literature value for 100% crystalline ETFE, which is 113.4 J/g, as reported by Walsby et al. in [27]. For the PE samples, it was not possible to determine the *T*_g_ because it is below −100 °C, which is outside the measurement range of the instrument used. Untreated ETFE has a slightly visible *T*_g_ at about 126 °C, but this disappears after ageing, probably due to an increased crystalline fraction and a lower amorphous fraction of the polymer.

### 3.2. Tensile Properties

**Figure 7:**
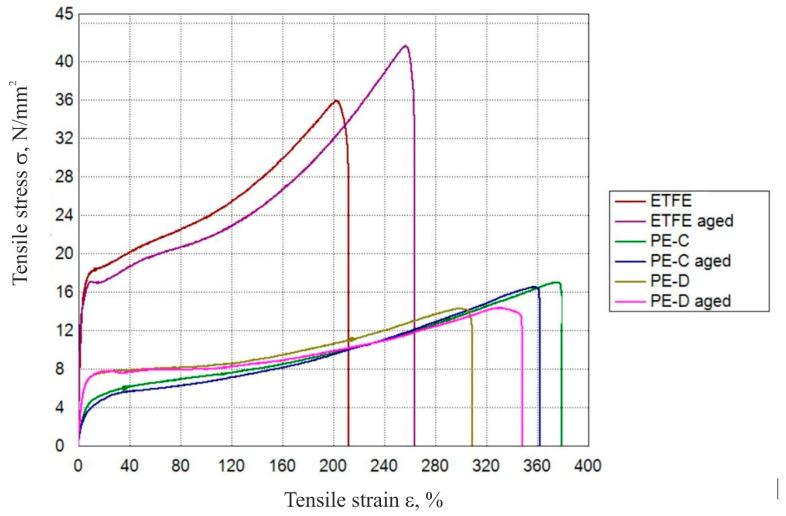
Comparison of the tensile properties of different plastic films during ageing.

Figure 7 shows a comparison of stress–strain curves for three types of plastic films before and after ageing. Additionally, Table 2 displays the values of tensile properties corresponding to each film type. Upon artificial ageing, the tensile strength and tensile modulus of aged diffuse PE film experienced a slight increase, measuring 2.7% and 5.4%, respectively. Furthermore, the tensile strain at break also increased by 12%. This stiffening phenomenon in polyethylene can be attributed to an increase in crystallinity, which consequently leads to the enhancement of tensile strength and modulus, as was indicated by Chabira et al. in their study [28] and supported by our DSC analysis in 3.1. Babaghayou et al. in their work also concluded that chemical and structural changes negatively affected some of the mechanical properties of the LDPE films, such as an increased modulus of elasticity because of the films stiffening during the weathering, as well as a decrease in elongation at break [11]. Dehbi observed that LDPE films experienced a decline in their mechanical properties because of exposure to environmental conditions, such as solar radiation and temperature. Solar radiation played an especially significant role in the degradation process. His study’s findings demonstrated a clear interrelation between the degradation of mechanical properties and the weathering and ageing processes [12].

In contrast, clear PE film exhibited a slight decrease in all three values: tensile strength, tensile modulus, and tensile strain at break, with reductions of 2.3%, 4.9%, and 5%, respectively. These findings correlate with Emekli et al.’s work [29], where they reported losses in tensile strength of LDPE films after 24 months of natural weather ageing between 3.3% and 8.5%. Additionally, they observed a decrease in tensile strain at break between 12% and 14% after the ageing period. In their study [2], Li et al. showed that the tensile strength of the PE film was adversely affected by two ageing methods: thermal ageing and ultraviolet ageing. Specifically, the tensile property decreased by 6% during thermal ageing, while it experienced a more substantial decline of 15% during ultraviolet ageing within 120 h. Clearly, the effects of ultraviolet ageing on the film’s tensile strength were more significant, leading to a faster deterioration of its mechanical properties.

On the contrary, the aged ETFE film displayed a notable 14.5% enhancement in tensile strength, along with a marginal 0.7% uptick in tensile modulus, a change attributed to the heightened crystallinity. Surprisingly, there was also an unforeseen 25% increase in tensile strain at break for the aged film. Conversely, Stefani et al. reported a slight decrease in tensile strength (6.4%) and tensile strain at break (7.1%) after 9800 h of artificial ageing at a constant dry temperature of 55 °C in their study [3]. Likewise, Bracciale et al. observed a slight decrease in tensile strength and tensile modulus of 5% in their work [17]. It is important to note that the variability in these reports can be attributed to the different ageing procedures and film formulations employed by each study.

The outcomes of the DSC analysis revealed that among the three films, notably PE-D and ETFE, there was an evident rise in crystallinity. This increase was reflected in higher values for both tensile strength and tensile modulus. However, unexpectedly, these films also displayed an increase in tensile strain at break, a behaviour that contrasts with the anticipated material response.

### 3.3. Transmittance

One of the critical factors that significantly influence covering materials is their ability to allow radiation permeability, particularly for photosynthetically active radiation (PAR) with high permeability. Light, which falls within the wavelength range of 380–760 nm, is an essential component of solar radiation. It plays a vital role in photosynthesis, the process by which plants convert solar energy into carbohydrates. Consequently, when purchasing a covering material, one of the most important considerations is its light transmission capability. Light transmission largely depends on the incident angle of the light and the reflections occurring on the surface of the covering material. The reflection is greatly influenced by both the angle of incidence and the material-specific index of refraction. Additionally, the material’s light transmission can vary depending on whether the incoming radiation is direct or diffuse. These considerations highlight the significance of selecting covering materials that allow appropriate levels of light penetration, ensuring optimal conditions for photosynthesis and the overall growth of plants [1]. The results showed that diffuse PE-D film had the lowest transparency, while clear PE-C film had a double level of transmitting light in the 400–1100 nm range compared to diffuse PE-D. All PE films demonstrated a significant decrease in transparency at wavelengths lower than 400 nm. These results correspond to the ones obtained by Abdel-Ghany et al. in [30], who subjected a new LDPE film to arid climatic conditions for a duration of one year and observed that the spectral properties of the film, along with its total transmittance to global and photosynthetically active radiation (PAR) solar radiation, decreased by approximately 32%. According to Stefani et al.’s findings, after artificial ageing for a duration of 9800 h in dry atmosphere, an ETFE film demonstrated a total transmission loss of approximately 1% for photosynthetically active radiation (PAR) in the range of 390 to 700 nm. [3]

**Figure 8:**
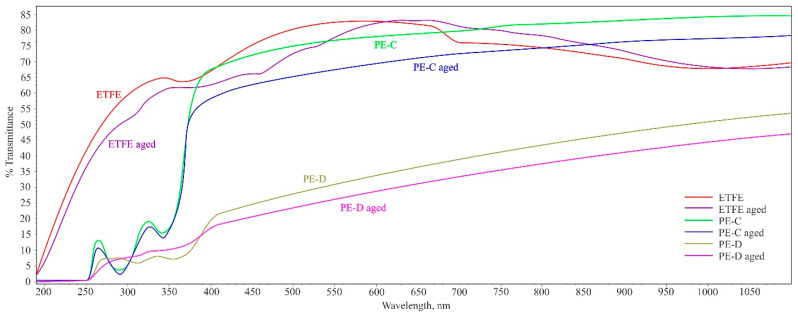
UV–VIS spectroscopy of plastic films (before and after ageing).

ETFE presented a comparable transmittance level to clear PE film and significantly higher transmittance in the region lower than 400 nm when compared to PE films of both types. Upon exposure to artificial ageing, there was an observed decrease in the degree of transmittance in all three types of films (Figure 8), which may indicate a certain degree of polymer degradation.

From these studies, it can be concluded that within the wavelengths that represent visible light (400 to 700 nm), ETFE film has transparency and transmits 70–95% of light both before and after exposure to ageing. Clear PE-C film has 60–75% of transmittance in the visible light range and diffuse PE-D film has only 20–35%. However, when moving into the UV light range (below 400 nm), PE films have a transmittance of 5–20%, while the ETFE film has values up to 65% transmittance, regardless of whether the films are exposed to atmospheric conditions or not. At wavelengths above 700 nm (IR light), both PE films exhibit an increase in transmittance, before and after exposure to ageing. Conversely, the ETFE film experiences a decrease in transmittance to 65% under the same conditions.

### 3.4. Chemical Structure Changes (FT-IR)

**Figure 9:**
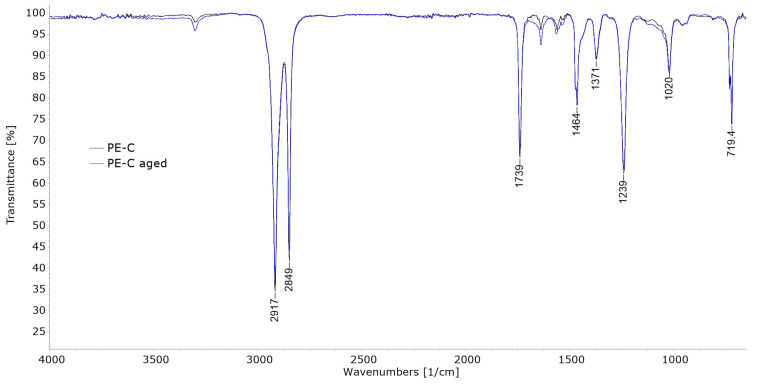
FT-IR spectra of PE-C.

**Figure 10:**
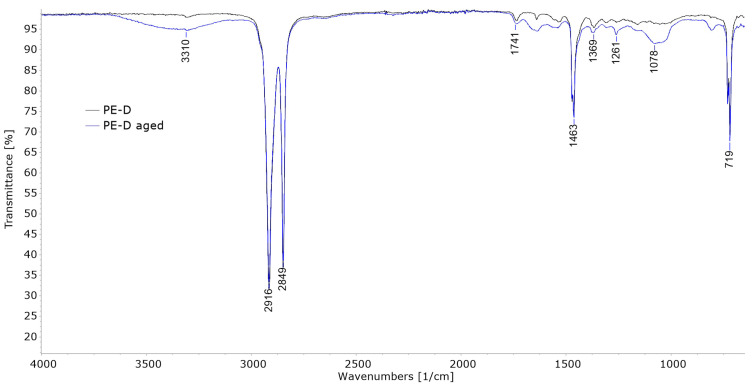
FT-IR spectra of PE-D.

**Figure 11:**
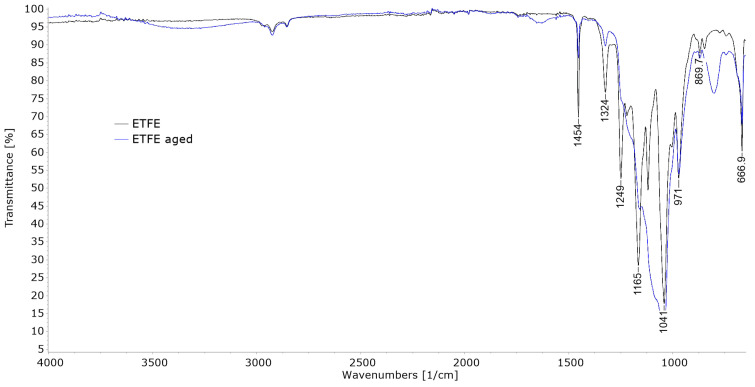
FT-IR spectra of ETFE.

FTIR analysis of the weathered films in the work of Babaghayou et al. [11] showed that exposure to sunlight promoted oxidation of the LDPE films, evident from increased carbonyl and vinyl indices. Figure 9, Figure 10 and Figure 11 show the comparison of FT-IR spectra of untreated and aged polymer film samples. Figure 9 shows the spectra of the sample PE-C. 

Apart from the typical C–H stretching and bending vibrations observed in polyethylene IR at 2917 cm^−1^, 2849 cm^−1^, 1464 cm^−1^, and 719 cm^−1^, as seen in the work of Renner et al. in [31], additional vibrations not commonly present in unused polyethylene can be identified. These include vibrations at 1739 cm^−1^, 1239 cm^−1^, and 1020 cm^−1^. The vibration at 1739 cm^−1^ is associated with the stretching of carbonyl C=O bonds, while the vibrations at 1239 and 1020 cm^−1^ are attributed to the stretching of C–O bonds, as reported by Setyaningrum et al. in [32]. The appearance of these bonds in polyethylene can be expected after degradation due to oxidation of the polymer chain but should not be present in an untreated sample. The presence can be explained either by an incorrect material designation by the supplier, when this type of polyethylene is actually a copolymer with another polymer containing C=O bonds, such as a type of acrylate, or by it containing an additive with C=O bonds. Possible additives used include different UV absorbers and stabilizers as well as antioxidants. It is well known that as UV stabilizers for polymers different hindered amine light stabilizers (HALS) are used, while antioxidants are usually sterically hindered phenols [33]. Both of these additives contain C=O bonds, while amines also contain N-H bonds. In the case of PE-C, beside the aforementioned vibrations, small but evident vibration at around 3250 cm^−1^ is also visible. This vibration can be attributed to N-H bonds in HALS [34], so this could explain the presence of bonds not characteristic for PE. The presence of some type of stabilizers would be expected in PE films for outdoor applications. The spectrum of aged PE-C is almost identical. There is no additional intensification of the C = O vibration, so the degradation of this sample was not visible using the FT-IR method.

Figure 10 shows the FT-IR spectra of the untreated and the aged PE-D sample. In this case, the polymer is indeed pure polyethylene, as it exhibits all the typical vibrations mentioned above. In this case, the spectrum changes slightly after ageing as new additional vibrations appear. The broad peak around 3300 cm^−1^ can be associated with –OH bond vibration and the weak vibrations below 1463 cm^−1^ could be associated with C–O stretching. All this is typical of the Norrish II type reaction of photooxidative degradation, as was explained by Gardette et al. in [35]. A small carbonyl C=O vibration is visible at 1741 cm^−1^ in both the untreated and aged samples, but the intensity does not increase significantly after degradation.

Figure 11 shows the FTIR spectra of the ETFE sample. It shows a very weak signal at 2978 cm^−1^ related to the C–H stretching vibration. Another signal at 1454 cm^−1^ relates to the deformation vibration of the –CH_2_ segment, while strong signals between 1324 cm^−1^ and 971 cm^−1^ relate to the C–F stretching and the signal at 667 cm^−1^ relates to the deformation vibration of the –CF_2_ segment. This corresponds to the findings of Yoo and Kwak et al. in [36] and Callela et al. in [37]. Significant changes are observed in the aged sample, as most of the signals in the 1324–971 cm^−1^ range turn into one major peak at 1041 cm^−1^, indicating significant changes in the structure of ETFE due to UV degradation.

### 3.5. DMA Thermograms

DMA thermograms were employed as graphical representations of the collected data to analyse the viscoelastic properties of the materials over a wide temperature range. Through these thermograms, essential parameters such as storage modulus and loss modulus (dynamic moduli) and tan δ were evaluated, providing insights into the materials’ mechanical behaviour and their response to dynamic loading and temperature variations. The storage modulus exhibits a direct relationship with the peak energy stored per cycle in the material (sample). Likewise, the loss modulus is proportionally related to the net energy dissipated per cycle. As the material undergoes ageing, these quantities experience changes, which, in turn, reveal the material’s ageing trend. Degradation, whether through chain scission or chain linking, results in alterations to the fundamental viscoelastic characteristics of the material. According to research of Kamweru et al., an elevation in dynamic modulus (increased stiffness) is associated with chain linking or crystallization, while a reduction in dynamic modulus indicates chain scission [38].

**Figure 12:**
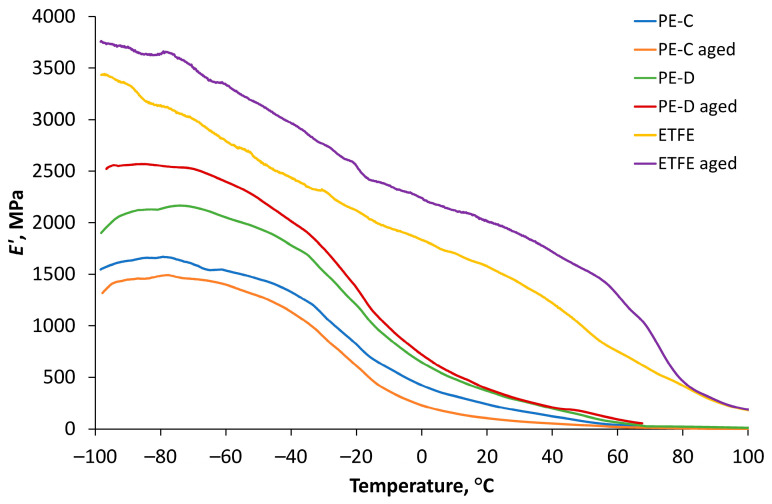
Storage modulus results for PE-D, PE-C, and ETFE films before and after ageing.

Figure 12 illustrates the storage modulus curves as a function of temperature for all films before and after ageing. The results indicate a significant decrease in the storage modulus of the clear PE film at all temperatures after ageing, with the highest values observed within the temperature range of −80 °C to −50 °C. On the other hand, for both diffuse polyethylene film and ETFE film, the values of storage modulus increased after ageing, possibly due to the formation of some cross-linked structures formed due to photo-degradation after ageing, which was also observed by FT-IR. Similar to the clear PE film, the largest storage modulus values for diffuse PE film and ETFE film were observed between −100 °C and −50 °C.

**Figure 13:**
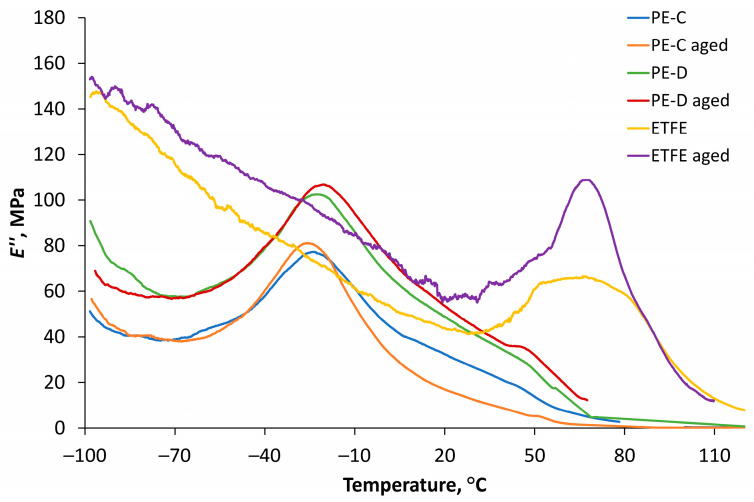
Loss modulus results for PE-D, PE-C and ETFE films before and after ageing.

Figure 13 presents DMA curves of loss modulus as a function of temperature for all films before and after ageing. In the case of the clear PE film before ageing at a temperature of −25 °C, a relaxation maximum, corresponding to molecular chain motion in the amorphous phase (beta peak), was observed. After ageing, the beta peak remains at the same temperature. For the diffuse PE film before ageing, the beta peak appeared at a temperature of −22.6 °C. However, after ageing, the beta peak shifted to a higher temperature (−20.1 °C), and there was an expansion of the relaxation maximum. This phenomenon can be attributed to changes occurring in the amorphous phase after ageing and the possible formation of a cross-linked structure, impeding the mobility of the polymer chain.

Regarding ETFE, the relaxation maximum was observed at a temperature of 64 °C, which shifted to a higher temperature (67.5 °C) after ageing. The most noticeable difference between aged and non-aged films was observed for ETFE, although ETFE films showed the highest values of loss modulus at almost all temperatures, except around the temperature range of −30 °C to −10 °C, where PE films curves reached the maximum value.

**Figure 14:**
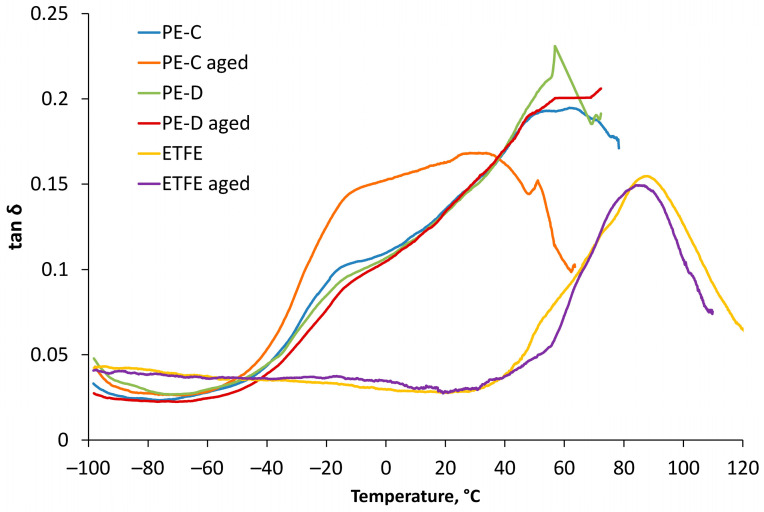
Tan δ results for PE-D, PE-C, and ETFE films before and after ageing.

Figure 14 displays DMA curves of tan δ as a function of temperature for all films before and after ageing. Tan δ serves as a measure of a material’s energy absorption and dissipation characteristics, representing the ratio of the loss modulus to the storage modulus.

Tan δ provides an essential insight into a material’s damping capabilities, with higher tan δ values indicating greater damping coefficients, thus representing a more efficient energy absorption and dissipation performance. In Figure 8, the tan δ curves clearly demonstrate that ETFE exhibits elastic behaviour at higher temperatures compared to polyethylene. This difference can be attributed to the distinct damping behaviours of the two materials, with polyethylene displaying higher tan δ peaks due to the dominance of the viscous component.

### 3.6. Thermogravimetric Analysis (TGA)

**Figure 15:**
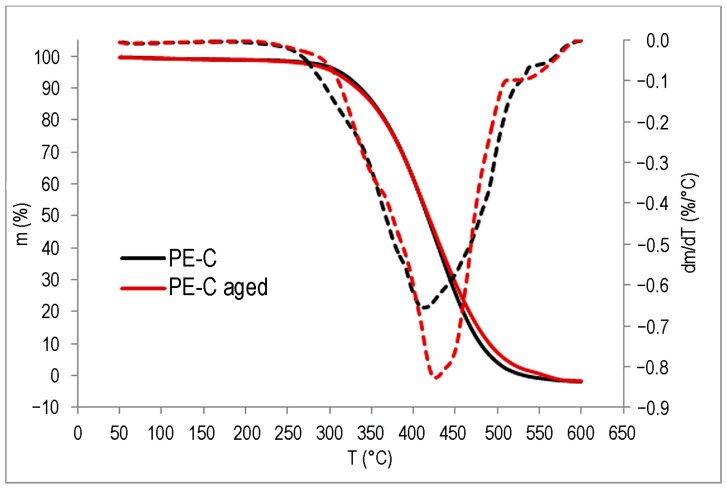
TG (**—**) and DTG (**– – –**) curves of PE-C.

**Figure 16:**
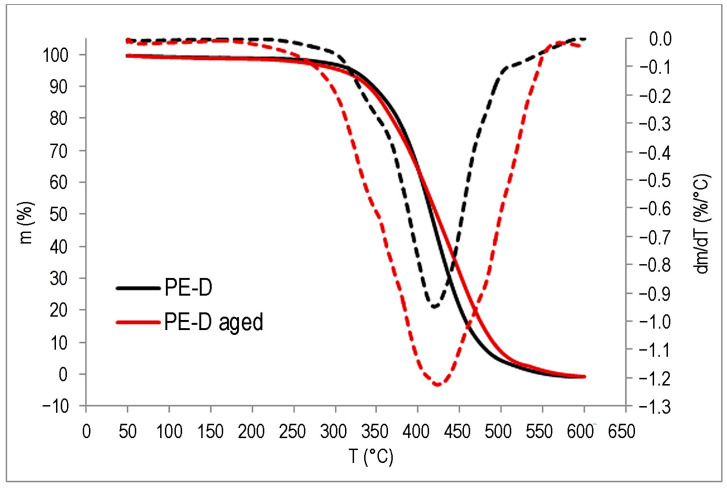
TG (**—**) and DTG (**– – –**) curves of PE-D.

**Figure 17:**
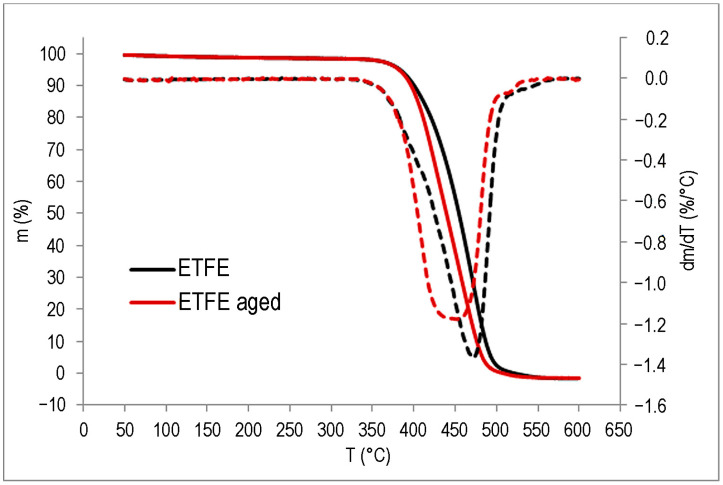
TG (**—**) and DTG (**– – –**) curves of ETFE.

The TGA results are shown in Figure 15, Figure 16 and Figure 17 and in Table 3.

The impact of UV degradation on the thermal stability of the polymers is evident; however, it affects each polymer in distinct ways. For the PE-C sample, degradation starts at a slightly higher temperature for the aged sample, but within the measurement uncertainty, while a clear difference can be seen for the *T*_max_, as the aged sample has a lower value by 8 °C. In addition, a second degradation step is possibly visible in the DTG curve (Figure 15) in the form of a small shoulder above 500 °C, supporting the possibility that another polymer component is present in the system, as suspected from the FT-IR. In the case of the PE-D sample, *T*_95_ is about 16 °C lower than the untreated sample, which is to be expected for a slightly degraded sample, as was found in the FT-IR analysis. Degradation results in a decrease in the molecular weight of the polymer, and volatile degradation products begin to appear at lower temperatures. The *T*_max_ values are slightly higher, but as can be seen from the DTG curve in Figure 16, all degradation occurs over a wider temperature range than for the untreated sample. The effects of degradation observed in FT-IR were also evident in TG analysis of the ETFE polymer, where degradation begins at similar temperatures for both samples, but the aged polymer has a lower *T*_max_ by about 12 °C, indicating lower thermal stability (Figure 17). Compared to PE samples, ETFE has much higher thermal stability as it belongs to the group of high-performance polymers. Degradation had no effect on the residual mass. All samples lost almost all their mass at 600 °C.

## 4. Conclusions

The study evaluated the chemical and mechanical stability of LDPE and ETFE greenhouse covering films after artificial ageing for 1000 h, following ISO-4892-2 [21] guidelines. The outcomes obtained from FT-IR, DMA, and TGA analyses are discussed to demonstrate the changes in both chemical and mechanical stability following the ageing process. The findings from both DMA and TGA analyses provided conclusive evidence that alterations occurred in all three plastic film specimens as a result of photo-degradation. This phenomenon was substantiated in the instances of PE-D and ETFE through FT-IR analysis, although no such confirmation was observed in the case of PE-C. 

Following the aging procedure, the transparent PE film experienced a slight decline in its tensile properties. This outcome was corroborated with DMA, which showed a decrease in storage modulus post-ageing. The clarity of the PE film slightly diminished, alongside a minor decline in light transmittance. FT-IR analysis indicated the presence of additives or copolymers in the film, which was proved using TGA. On the other hand, the diffuse PE-D film experienced an improvement in tensile properties, likely attributed to cross-linking caused by photo-oxidation during ageing, which was supported through DMA, which showed an increase in storage modulus. Also, there was a slight drop in transmittance. DSC results showed an increase in crystallinity. Degradation was proved via TGA and FT-IR.

Just like the diffuse PE-D film, the ETFE film displayed enhanced tensile properties resulting from cross-linking. This phenomenon was also apparent through DMA analysis, which exhibited an elevation in the storage modulus. Concurrently, the loss modulus demonstrated a decrease as revealed with DMA, while DSC measurements indicated an increase in crystallinity. The influences of degradation were evident in FT-IR analysis, as well as in TGA results. Notably, TGA findings also highlighted the superior thermal stability of ETFE compared to polyethylene films.

Among the examined samples, the lowest increase in crystallinity was observed in the PE-C sample. This could be attributed to the potential presence of UV stabilizers, as identified with FT-IR analysis. Conversely, the minor rise in crystallinity in this sample resulted in a less pronounced stiffening effect, and consequently, a marginal reduction in tensile properties.

The findings provide valuable insights for optimizing the selection of greenhouse coverings to ensure long-term performance and efficiency in agricultural settings.

Overall, the study demonstrates that while all three films experience some degree of property changes due to ageing, their degradation remains within acceptable limits for greenhouse coverings. ETFE film, with its superior properties, emerges as a particularly promising option, especially in scenarios requiring high transmittance. Future studies could explore the long-time performance of these films across diverse environmental scenarios, along with potential approaches to alleviate any observed degradation.

## Figures and Tables

**Table 1:** Characteristic DSC parameters of studied samples; glass transition temperature (*T*_g_), melting temperature (*T*_m_), melting enthalpy (Δ*H*_m_), crystallization degree (*X*_c_), crystallization temperature (*T*_c_), and crystallization enthalpy (Δ*H*_c_).

Sample	*T*_g_ (°C)	*T*_m_ (°C)	Δ*H*_m_ (J/g)	*X*_c_ (%)	*T*_c_ (°C)	Δ*H*_c_ (J/g)
PE-C	/	94.3	52.1	17.8	79.2	61.5
PE-C aged	/	94.4	56.7	19.4	79.2	58.0
PE-D	/	105.6	63.4	21.6	105.1	60.9
PE-D aged	/	109.3	74.5	25.4	96.4	66.5
ETFE	126.4	253.7	22.1	19.5	239.6	23.3
ETFE aged	/	253.2	29.0	25.6	240.0	23.3

**Table 2:** Tensile properties of PE-D, PE-C, and ETFE films before and after ageing.

	Tensile Strength *σ*_m_, N/mm^2^	Tensile Strain at Break *ε*_b_, %	Tensile Modulus *E*, N/mm^2^
ETFE—average	37.2 ± 8.5	208.0 ± 80.7	996.0 ± 60.2
ETFE aged—average	42.6 ± 2.2	260.3 ± 16.8	1003.0 ± 145.7
PE-C—average	17.2 ± 6.2	376.5 ± 128.5	84.0 ± 3.7
PE-C aged—average	16.8 ± 9.4	357.7 ± 223.5	79.9 ± 18.7
PE-D—average	14.7 ± 5.5	303.9 ± 122.3	233 ± 15.1
PE-D aged—average	15.1 ± 4.6	341.4 ± 122.5	246 ± 14.8

**Table 3:** Temperature at 5 mass% loss (*T*_95_), temperature at maximum degradation rate (*T*_max_) and residual mass at 600 °C (*m*_600_).

Sample	*T*_95_ (°C)	*T*_max_ (°C)	*m*_600_ (%)
PE-C	321.5	445.4	0.1
PE-C aged	323.5	437.5	0.2
PE-D	327.6	428.0	0.2
PE-D aged	311.8	432.9	0.1
ETFE	383.0	470.0	0.2
ETFE aged	381.8	457.6	0.2

## Data Availability

The data presented in the study are available on request from the corresponding author.
